# How far from the gold standard? Comparing the accuracy of a Local Position Measurement (LPM) system and a 15 Hz GPS to a laser for measuring acceleration and running speed during team sports

**DOI:** 10.1371/journal.pone.0250549

**Published:** 2021-04-23

**Authors:** Karin Fischer-Sonderegger, Wolfgang Taube, Martin Rumo, Markus Tschopp

**Affiliations:** 1 Swiss Federal Institute of Sport Magglingen SFISM, Section for Elite Sport, Magglingen, Switzerland; 2 Department of Neurosciences and Movement Science, University of Fribourg, Fribourg, Switzerland; University of Cassino e Lazio Meridionale, ITALY

## Abstract

**Purpose:**

This study compared the validity and inter- and intra-unit reliability of local (LPM) and global (GPS) position measurement systems for measuring acceleration during team sports.

**Methods:**

Devices were attached to a remote-controlled car and validated against a laser. Mean percentage biases (MPBs) of maximal acceleration (a_max_) and maximal running speed (v_max_) were used to measure validity. Mean between-device and mean within-device standard deviations of the percentage biases (bd-SDs and wd-SDs) of a_max_ and v_max_ were used to measure inter- and intra-unit reliability, respectively.

**Results:**

Both systems tended to underestimate a_max_ similarly (GPS: –61.8 to 3.5%; LPM: –53.9 to 9.6%). The MPBs of a_max_ were lower in trials with unidirectional linear movements (GPS: –18.8 to 3.5%; LPM: −11.2 to 9.6%) than in trials with changes of direction (CODs; GPS: –61.8 to −21.1%; LPM: −53.9 to –35.3%). The MPBs of v_max_ (GPS: –3.3 to –1.0%; LPM: –12.4 to 1.5%) were lower than those of a_max_. The bd-SDs and the wd-SDs of a_max_ were similar for both systems (bd-SDs: GPS: 2.8 to 12.0%; LPM 3.7 to 15.3%; wd-SDs: GPS: 3.7 to 28.4%; LPM: 5.3 to 27.2%), whereas GPS showed better bd-SDs of v_max_ than LPM.

**Conclusion:**

The accuracy depended strongly on the type of action measured, with CODs displaying particularly poor validity, indicating a challenge for quantifying training loads in team sports.

## Introduction

Team sports are characterized by frequent changes of direction (CODs) and many accelerations and decelerations [1, 2]. Since accelerating consumes more energy than maintaining a constant speed, measuring the distance achieved at different speeds does not completely reflect training or game loads [1, 3–5]. Therefore, in order to assess short, intense actions that are typical of team sports, position measurement systems that can accurately measure acceleration are needed [1]. Both global (GPS) and local (LPM) position measurement systems have been used to this end [6–8]. GPS uses satellites to determine the positions of players; it is therefore not locally bound and can be used flexibly across different sites (e.g., home and away games, training and match fields). However, the number of available satellites can influence GPS’s accuracy [9]. Satellites may also be hidden or have their signals reflected by physical obstacles such as buildings or trees, and can furthermore be affected by atmospheric conditions. In contrast, the LPM system uses base stations located around a specific site [7]. Its use is therefore limited to a fixed location, but is not affected by the satellite limitations of GPS and has a higher sampling frequency. We therefore hypothesized that the LPM measurement accuracy would be higher than GPS, particularly when measuring athletes’ movement patterns in team sports.

Despite the importance of measuring accelerations to assess physical load, most studies that have sought to validate position measurement systems in team sports have compared running distances [6, 7, 10–12] or averaged speed [8, 11, 13, 14] to a gold standard. For total distance, the measurement error of three different 1 Hz GPS devices was established to be < 5% [6] and the coefficient of variation (CV) for 10 Hz and 18 Hz GPS ranged from 2.5 to 13.0% and 1.1 to 5.1%, respectively [10]. Similarly, Frencken et al. [7] used total distance in a team sport context to validate an LPM system’s accuracy and found that it generally underestimated actual distance by up to –1.6% and mean speed by –0.1 to −0.6 km·h^−1^. However despite fairly low error indicators for both systems, the key movements of team sports, such as accelerations and changes of direction, cannot be captured by such measurements [15]. Previous studies have accounted for accelerations when validating the accuracy of position measurement systems. Akenhead et al. [16] demonstrated that the validity and reliability of speeds measured with a 10 Hz GPS were inversely correlated to acceleration—i.e., the higher the acceleration, the lower the validity and reliability of speed measurements during the acceleration. Furthermore, Buchheit et al. [17] compared three different GPS models and showed a between-unit variation in peak acceleration of 10%; and the CV for maximal speed and maximal acceleration in Lacome et al. [18] was 0.5% and 6.4% respectively, as measured during a 40 m sprint test. However, to our knowledge, no studies have investigated accelerations during changes of direction.

While distances can be validated with a trundle wheel or measuring tape and averaged speeds can be measured with timing gates [6, 19, 20], there is no “gold standard” to assess acceleration in team sport-specific actions. Ideally, such a system should be able to measure both speed and acceleration, since the ability to accelerate depends on the speed at baseline. More specifically, maximal voluntary acceleration has been shown to decrease with increased initial running speed [21]. Lasers and radar beams are therefore more suitable measurement tools than timing gates [19, 22–24]. When used with adequate signal filtering, lasers are understood to accurately measure speed and acceleration [25], with an average speed error of < 2%, as reported by Tuerk-Noack and Schmalz [26]. Another study found that the typical error when using lasers to measure speed in a repeated running trial was very small (0.05 m·s^–1^) and that the intra-class correlation was high (*r* = 0.98) [27]. However, it is also known that laser measurements’ accuracy is limited during the first acceleration phase of a sprint due to shifts in the human body’s center of gravity [28]. Furthermore, an athlete’s upper-body movements during a sprint can negatively influence the accuracy of the laser [16, 29, 30]. These limitations can be circumvented if the movement of the human body is simulated by a rigid body, such as a vehicle. Additionally, a vehicle offers the opportunity to wear several devices at the time. However, to date, only three GPS accuracy studies have analyzed acceleration using an object other than a person as a device carrier [16–18].

To determine the inter-unit reliability of several GPS and LPM devices, and less so the intra-unit reliability, a number of devices should be attached to the same person or object. Since there is limited space to adequately position multiple measurement devices on the back of a human, most studies to date have only used two to four devices to avoid encountering problems (e.g., discomfort, movement restrictions, additional weight) [11, 24, 31]. Other studies have approached this problem by attaching only one device at a time to an athlete’s back and then instructing the athlete to complete a course several times [13, 32, 33]; however, this approach cannot differentiate between measurement errors of the different devices versus changes in the athlete’s movement execution.

So far, no studies have assessed positioning system accuracy using a non-human object as a device carrier, while simultaneously comparing the accuracy of two different systems (i.e., global and local positioning systems) to a gold standard in different team sport-specific actions. Thus, the aim of this study was to determine and compare the validity of commercially available GPS and LPM systems in assessing acceleration and speed during forward and backward and single directional actions, using a laser measurement system as gold standard. Furthermore, this study aimed to assess the inter-unit and intra-unit reliability of GPS and LPM speed and acceleration measurements in different team sport-specific actions.

## Materials and methods

### Testing procedures

To examine the validity and the inter- and intra-unit reliability of a 15 Hz GPS and an LPM system, a remote-controlled car (RCC) (Traxxas Rally rushless 4WD 1/10 RTR, Model 70, Plano, Texas), steered by an operator, was used to carry the position measurement devices and simulate movement patterns common to team sports. One team sport-specific movement pattern can provide several team sport-specific actions (e.g. a movement pattern with two accelerations interspersed with an abrupt deceleration can be accounted for both low acceleration from standstill [LA] and acceleration after an abrupt deceleration [A-D]). Team sport-specific actions differing in acceleration capacity, initial speed, maximal speed, number of CODs, and were divided into seven subcategories. In order to present a number of trials within each subcategory (Table 1), sport-specific movement patterns were carried out several times. Throughout, the operator attempted to achieve speeds and accelerations that mimicked team sport-specific movement patterns as closely as possible. Accelerations occurred between 0 and 7.8 m·s^−2^, and were classed as low acceleration if < 3.5 m·s^−2^ or high acceleration if > 3.5 m·s^−2^. Flying start accelerations were initialized from speeds between 6 and 15 km·h^−1^. This approach allowed us to identify differences in validity and inter- and intra-unit reliability of maximal speed (v_max_) and maximal acceleration (a_max_) during different types of actions.

**Table 1:** Subcategories of different team sport-specific actions and the number of trials within the subcategory.

Subcategory	# of trials
low acceleration from standstill (LA)	8
high acceleration from standstill (HA)	6
high acceleration from a flying start (HA-flyingS)	6
acceleration after an abrupt deceleration (A-D)	4
acceleration after a 180° change of direction (A-COD)	4
repetitive high acceleration, shuttle runs 4 x 5m (RA-5m)	6
repetitive high acceleration, shuttle run 4 x 10m (RA-10m)	6

**Fig 1:**
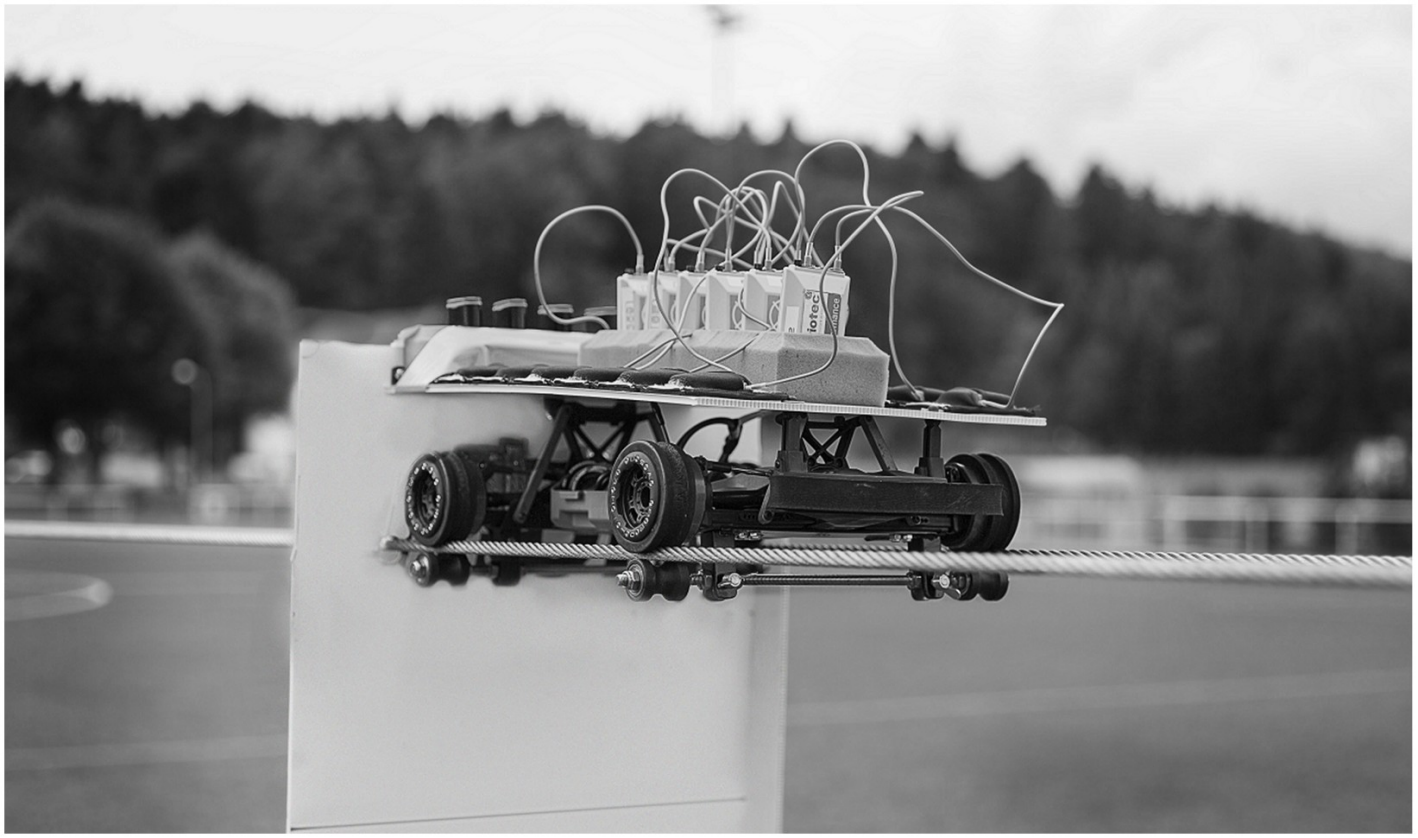
Remote-controlled car (RCC) driving on two steel ropes. Six LPM and six GPS devices were attached on the top of the RCC. At the back of the RCC was a reflective, white panel used to detect the laser beam.

Six GPS devices (15 Hz GPS, SPI HPU, GPSports Pty Ltd, Canberra, Australia) and six LPM devices (Inmotiotec GmbH, Regau, Austria) were simultaneously mounted in an upright position on a platform installed on top of the RCC. Care was taken to ensure that the LPM systems’ antennas were horizontal, as they are when attached to an athlete’s shoulder, and that all antennas were free from obstructions. The RCC’s wheels were modified to allow it to drive on two steel ropes stretched 40 cm apart and 1.50 m above the ground, simulating the height at which position measurement devices are normally worn when attached to athletes (Fig 1). The steel ropes were stretched across the entire length of the soccer field through the middle of the field. The actions were carried out in the middle of the field.

The raw GPS data was interpolated by the device’s software from 5 Hz to 15 Hz, as in Nagahara et al. [34]. An LPM system can record position data up to 1,000 Hz, but the effective frequency for each device is divided by the number of devices that are used. To reach the same frequency as in official games, 16 additional devices were randomly placed across the field, for a total of 22 devices. Thus, each LPM device recorded at a sampling frequency of 45 Hz (1,000 / 22). No trees or buildings were positioned around the measurement site, and testing was conducted under a clear blue sky with no cloud cover.

All collected GPS and LPM data were downloaded using customized software (SPI: Team AMS51; GPSports Systems, Canberra, Australia; LPM: Inmotio software, Inmotiotec GmbH, Regau, Austria) and then exported to Microsoft Excel (2016). The GPS and LPM customized software programs both provided speed and acceleration data by default. Validity and inter- and intra-unit reliability of speed and acceleration were tested by concentrating on v_max_ and a_max_ from each trial within a subcategory, which were manually extracted in a post-processing step.

A laser (LDM301, Jenoptic, Jena, Germany) with a sampling frequency of 100 Hz was used as the criterion to measure speed and acceleration of the RCC during both forward and backward and single directional movement patterns. The use of the RCC favored to ensure a complete laser signal, as a player’s upper body can quickly shift outside the laser’s range during a sprint compared to the car, which drives in a straight line with no lateral deviations. As a laser measures the time delay of a reflected pulsed infrared light, the sighting beam was focused on the center of a 0.3 m x 0.5 m white reflective panel secured on the RCC; the laser was located on a tripod 3m behind the start. Raw data was collected using the respective manufacturer software and then exported to Microsoft Excel (2016). During data post-processing, the first and second derivatives of the position data from the laser were calculated to obtain the speed and acceleration data. Subsequently, a moving average filter was used over 20 data points (forward and backward) to smooth the speed and acceleration data.

### Statistical analysis

The v_max_ and a_max_ means and between-device standard deviations (SDs) measured by GPS and LPM devices, as well as the v_max_ and a_max_ means measured by the laser, were assessed for each subcategory of the team sport-specific actions (Table 1). To evaluate the validity of the two position measurement systems, the mean percentage biases (MPBs) of a_max_ and v_max_ for GPS and LPM were expressed relative to the laser (gold standard), using the following formula: (100 × GPS parameter / laser parameter– 100) and (100 × LPM parameter / laser parameter– 100) (similar to Nagahara et al. [34]). The MPBs were calculated for each trial and averaged for each subcategory.

Similarly, to assess inter-unit reliability, between-device SDs of the percentage biases of a_max_ and v_max_ for all six devices were calculated for each trial and averaged for each subcategory. Additionally, the typical error was calculated and expressed as a CV.

For intra-unit reliability, the within-device SD of the percentage biases of a_max_ and v_max_ for each individual device was calculated over all trials and averaged for each subcategory. Both the between- and within-device SDs of the percentage biases, representing the inter- and intra-unit reliability, respectively, were expressed as a percentage relative to the laser-derived values. This approach was chosen because of the inevitable variations in the levels of acceleration and maximum speed between different trials within each subcategory. Using the SD of the absolute value instead of the SD of the percentage bias—especially when determining the intra-unit reliability—would have led to a misinterpretation of the reliability, due to the RCC’s varying acceleration and speed curves from trial to trial.

## Results

### Validity of maximal acceleration

**Fig 2:**
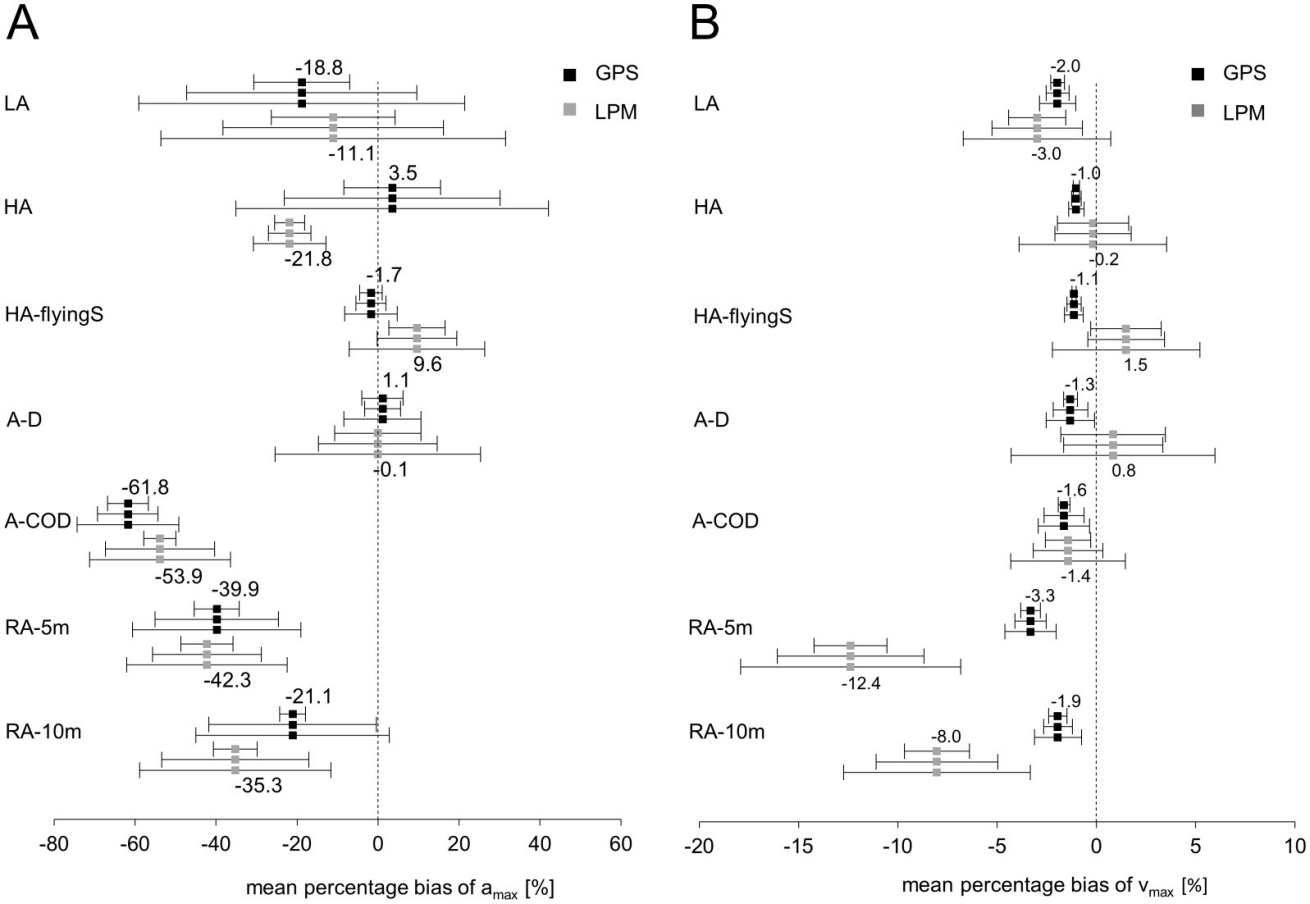
Validity, inter- and intra-unit reliability of maximal acceleration (a_max_; A) and maximal speed (v_max_; B). Mean percentage bias (MPB; relative bias of the GPS and LPM devices compared to the laser) of a_max_ and v_max_ of 7 different team sport-specific actions indicates the validity of a_max_ and v_max_. The first whisker of each subcategory within the black and grey squares is the between-device SD of the percentage biases of a_max_ and v_max_, and represents the inter-unit reliability of a_max_ and v_max_. The second whisker is the within-device SD of the percentage biases of a_max_ and v_max_, and represents the intra-unit reliability of a_max_ and v_max_. The third whisker of each subcategory is the combination of the between- and within-device SD of the percentage biases of a_max_ and v_max_, which indicates the inter- and intra-unit reliability of a_max_ and v_max_. If a GPS or LPM device is randomly chosen for repeated measures, the third whisker illustrates the percentage measurement error.

Table 2 shows the means and between-device SDs of a_max_ during different team sport-specific actions as measured by the GPS and LPM systems, and the mean of the laser measurement. Fig 2A shows the MPB of a_max_ as an indicator of validity. Both the GPS and the LPM devices tended to underestimate a_max_, especially for actions that included CODs. The exceptions were high acceleration from standstill (HA) and A-D, which were slightly overestimated by GPS (MPB: 3.5% and 1.1%, respectively) and high acceleration from a flying start (HA-flyingS), which was overestimated by LPM (MPB: 9.6%). Moreover, the MPBs for a_max_ were lower for both systems when measuring linear movements (LA, HA, HA-flyingS, and A-D) as compared to actions with CODs (acceleration after a 180° change of direction [A-COD], repetitive high acceleration, shuttle runs 4 x 5m [RA-5m], and repetitive high acceleration, shuttle runs 4 x 10m [RA-10m]) and were higher for RA-5m than for RA-10m. The highest MPB was in A-COD (GPS: –61.8%; LPM: –53.9%).

**Table 2:** Means and between-device standard deviations (SDs) of maximal acceleration (a_max_) during different team sport-specific actions measured with a GPS and an LPM system and the mean of a_max_ measured with laser.

Subcategory	Laser (m·s^−2^)	GPS			LPM		
		Mean (m·s^−2^)	SD (m·s^−2^)	CV (%)	Mean (m·s^−2^)	SD (m·s^−2^)	CV (%)
low acceleration from standstill (LA)	1.76	1.32	0.22	16.1	1.49	0.22	14.7
high acceleration from standstill (HA)	6.25	6.55	0.79	10.3	4.90	0.24	4.8
high acceleration from a flying start (HA-flyingS)	4.62	4.54	0.13	2.8	5.06	0.32	6.3
acceleration after an abrupt deceleration (A-D)	2.85	2.85	0.13	5.0	2.99	0.25	11.1
acceleration after a 180° change of direction (A-COD)	3.46	1.33	0.18	12.8	1.65	0.14	8.7
repetitive high acceleration, shuttle runs 4 x 5m (RA-5m)	5.08	3.04	0.29	9.8	2.94	0.32	10.7
repetitive high acceleration, shuttle runs 4 x 10m (RA-10m)	5.16	4.25	0.16	4.4	3.44	0.27	9.3

The Coefficient of Variation (CV) is an indicator of the inter-unit reliability.

### Validity of maximal running speed

**Fig 3:**
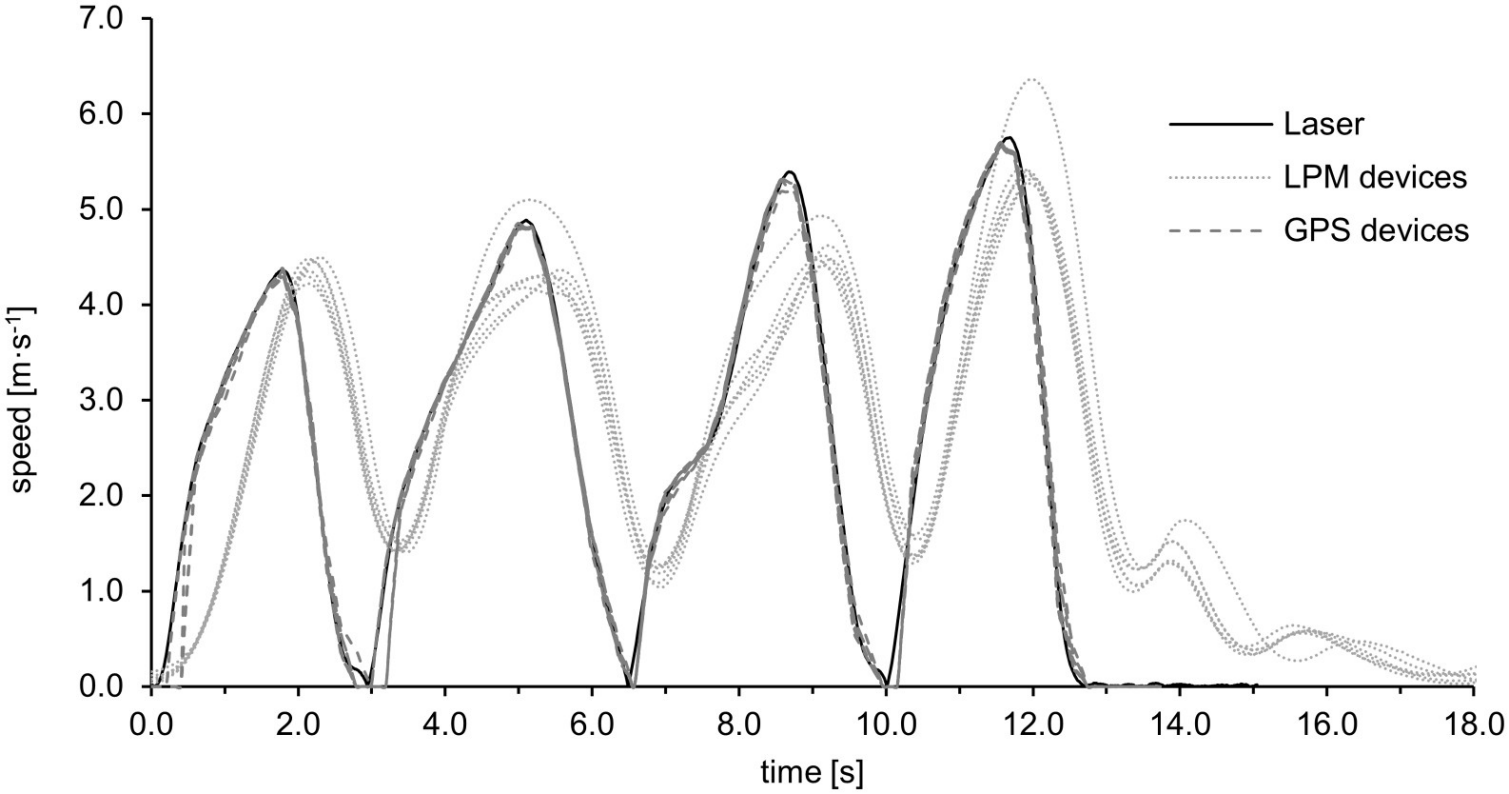
Speed curve of the laser signal, 6 GPS and 6 LPM signals during repetitive high-accelerated shuttle runs 4 x 5m (RA-5m).

Table 3 shows the means and between-device SDs of a_max_ during different team sport-specific actions as measured by the GPS and LPM systems, and the mean of the laser measurement. The MPB of v_max_ is presented in Fig 2B as an indicator of validity. In general, the MPBs of v_max_ were markedly lower than of a_max_; similar to a_max_, v_max_ tended to be systematically underestimated by GPS (−3.3 to −1.0%), whereas both negative and positive biases occurred with LPM (–12.4 to 1.5%). The largest differences between the GPS and LPM measurements were in RA-5m (GPS: –3.3%; LPM: –12.4%) and RA-10m (GPS: –1.9%; LPM: –8.0%). As shown in Fig 3, the LPM speed curve was clearly time-delayed during these repetitive accelerations, and its maximum recorded speed was considerably lower than the speed measured with the laser and GPS (with the exception of one LPM device in the second and fourth CODs).

**Table 3:** Means and between-device standard deviations (SDs) of maximal running speed (v_max_) during different team sport-specific actions measured with a GPS and an LPM system and the mean of a_max_ measured with laser.

Subcategory	Laser (m·s^−1^)	GPS			LPM		
		Mean (m·s^−1^)	SD (m·s^−1^)	CV (%)	Mean (m·s^−1^)	SD (m·s^−1^)	CV (%)
low acceleration from standstill (LA)	6.06	5.95	0.02	0.36	5.86	0.08	1.48
high acceleration from standstill (HA)	10.05	9.95	0.01	0.63	10.04	0.19	1.78
high acceleration from a flying start (HA-flyingS)	11.04	10.92	0.01	0.11	11.21	0.20	1.76
acceleration after an abrupt deceleration (A-D)	7.21	7.13	0.02	0.36	7.31	0.19	2.62
acceleration after a 180° change of direction (A-COD)	5.42	5.34	0.01	0.30	5.33	0.06	1.15
repetitive high acceleration, shuttle runs 4 x 5m (RA-5m)	4.79	4.63	0.02	0.51	4.20	0.09	5.44
repetitive high acceleration, shuttle runs 4 x 10m (RA-10m)	7.05	6.91	0.03	0.47	6.46	0.12	3.25

The Coefficient of Variation (CV) is an indicator of the inter-unit reliability.

### Inter- and intra-unit reliability of maximal acceleration

The inter-unit and intra-unit reliability of a_max_, as measured by both GPS and LPM, is shown in Fig 2A as the between-device and within-device SDs of the percentage biases. Table 2 shows the CV of the GPS and LPM measurements, which can be also interpreted as an indicator of inter-unit reliability.

The inter-unit reliability of a_max_, as measured by GPS, ranged from 2.8 to 5.6% (expressed as the between-device SDs) for most of the tested actions, with the exceptions of LA (11.8%) and HA (12.0%). For LPM, the inter-unit reliability of a_max_ ranged from 4.0 to 7.0%, with the exceptions of A-D (10.7%) and LA (14.6%).

The intra-unit reliability of a_max_, as measured by GPS, ranged from 3.7 to 28.4% (expressed as the within-device SDs). Accelerations initiated from a standstill (HA and LA) had the lowest intra-unit reliability (26.6% and 28.4%, respectively), while HA-flyingS and A-D had the highest (3.7% and 4.4%, respectively). For LPM, the intra-unit reliability of a_max_ ranged from 5.3 to 18.2%, with the exception of LA (28.3%); HA and HA-flyingS had the highest intra-unit reliability (5.3% and 9.8%, respectively).

For GPS, the highest inter- and intra-unit reliability of a_max_—i.e., its summarized reliability—was found for HA-flyingS and A-D (6.5% and 9.5%, respectively), while HA and LA (38.6% and 40.3%, respectively) displayed the lowest summarized reliability. For LPM, HA generated the highest summarized reliability (9.0%) and LA the lowest (43.0%). The summarized reliability illustrates the percentage measurement error if a GPS or LPM device is randomly chosen for repeated measures.

### Inter- and intra-unit reliability of maximal running speed

Fig 2B shows the inter- and intra-unit reliability of v_max_ measured by both GPS and LPM as between-device and within-device SDs of the percentage biases.

The overall inter- and intra-unit reliability of v_max_ was considerably higher than that of a_max_. The inter-unit reliability values for v_max_ were all smaller than 0.5% when measured by GPS and smaller than 2.6% when measured by LPM, across all subcategories.

Furthermore, the *intra*-unit reliability of v_max_ was lower than the *inter*-unit reliability for both systems, with the sole exception of A-D when measured by LPM. The intra-unit reliability was lower using LPM than GPS (GPS: between 0.2% and 1.0% and LPM: between 1.8% and 3.7%). Moreover, the GPS summarized reliability values for v_max_ were below 1.3% across all subcategories, while the LPM summarized values for v_max_ ranged from 2.9 to 5.6%.

## Discussion

The present study examined validity and inter- and intra-unit reliability of two different position measurement systems (local and global), when evaluating the accuracy of acceleration measurements in different team sport-specific actions. In contrast to our hypothesis, the current data revealed that in most cases the validity of a_max_ in team sport-specific actions was not lower when measured with the 15 Hz GPS than with the LPM system. However, both systems’ a_max_ results demonstrated surprisingly large discrepancies compared to the “gold standard” laser measurements. On the other hand, the validity of v_max_ was generally good and comparable to the “gold standard” in both systems.

Inter-unit reliability of a_max_ was similar across both systems, while inter-unit reliability of v_max_ was noticeably better with the GPS than with the LPM system. The inter-unit reliability of a_max_ and v_max_ was generally better than the intra-unit reliability using both measurement systems.

### Validity of the acceleration assessment

Overall, the results show that the validity for assessing a_max_ using both systems is lower than the validity for assessing v_max_. Although acceleration is the derivative of speed over time and it could be assumed that the a_max_ biases would correlate to the v_max_ biases, it must be noted that the a_max_ values were attained earlier than the v_max_ values in every action [21]. For this reason, the a_max_ measurement errors did not correlate with the v_max_ ones. Furthermore, validity of a_max_ (and v_max_) not only depended on the type of measurement system, but also on the type of action being evaluated: both systems demonstrated the lowest validity of a_max_ during actions with one COD (A-COD), and validity remained low in actions with multiple CODs (RA-5m, RA-10m). Furthermore, validity of a_max_ was higher when high-accelerated actions are initiated from a flying start (HA-flyingS) than from standstill (LA, HA). The reason for the great difference in validity of a_max_ between accelerations from standstill and from a flying start may be due to manufacturer’s filter settings which smoothed the raw data [35, 36]. The Kalman filter explicitly relies on predictions of the next measurement and therefore assumes predictable trajectories. However, keeping trajectories unpredictable is an important factor in team sports. As shown in Fischer-Sonderegger et al. [37], soccer players are often already in motion before high acceleration occurs, but also accelerate from standstill. Consequently, the MPBs of a_max_ can vary greatly depending on the type of action or type of exercise. For example, MPBs are higher in small-sided games, where unpredictable CODs often occur, and lower during constant running drills that lack abrupt changes in direction or speed. Nonetheless, it is worth noting that validity of a_max_ for actions with high accelerations, both from a standstill and from a flying start, were noticeably better when measured with the GPS than with the LPM system.

As recognized above, both measurement systems had the lowest validity of a_max_ when accelerations occurred after an abrupt 180° COD (A-COD). During the trial with multiple CODs within 10 m (RA-10m), LPM had higher MPBs in a_max_ than GPS. When the running distance between the CODs was reduced to 5 m (RA-5m), the validity of a_max_ decreased further and was equally poor for both systems (–39.9% for GPS and –42.3% for LPM). As shown in Fig 3, LPM’s poor ability to assess abrupt CODs is illustrated by its incomplete deceleration curves prior to the next acceleration: even if the effective speed was 0 for a few tenths of a second during the COD, the measured speed, and therefore also the measured acceleration, never reached 0. This led to a considerably lower validity for LPM a_max_ values when several CODs occurred within short time intervals and without long standstill phases. As mentioned previously, we assume that these incomplete deceleration curves were due to the use of Kalman filters with inadequate predictions [35, 36].

In addition, when acceleration occurred after an immediate deceleration but without a COD (A-D), the validity of a_max_ was noticeably better than in trials with one or more CODs (A-COD, RA-5m, and RA-10m) and was relatively strong for both systems (GPS: 1.1%; LPM: –0.1%). One possible explanation for the large difference in validity of a_max_ between A-D and A-COD, RA-5m, and RA-10m could be due to the Kalman filter settings. In contrast to A-COD, RA-5m, and RA-10m, the direction of A-D is already correctly predicted by the Kalman filter. This leads to the difference in validity between a stop-and-go with COD (A-COD, RA-5m and RA-10m) compared to without COD (A-D). However, this can only be speculated and would have to be verified in further studies with different Kalman filter settings. The chosen test setting allowed only the testing of the two extremes of a stop-and-go action, represented in the subcategories A-D versus A-COD, RA-5m, and RA-10m.

### Validity of speed assessment

We found that the GPS systematically underestimated v_max_; the MPBs of v_max_ ranged from –3.3 to −1.0%. These results are comparable to those of Lacome et al. [18], who found an overall bias of –3.0%. Buchheit et al. [32] observed a positive mean bias of v_max_ of 0.3% for 5 Hz GPS measurements. However, no abrupt 180° CODs were performed which produced the highest negative biases in our study. In contrast, the biases of v_max_ measured by the LPM system were unsystematic and ranged from –12.4 to 1.5%. Again, the results of this study are poorer than the results of Buchheit et al. [32], who measured a mean bias of v_max_ of 0.4% for LPM measurements. Similar to a_max_, validity of v_max_ was markedly lower in actions with multiple CODs than in linear movements: for GPS, the highest bias was –3.3% (RA-5m), while for the LPM system, the bias reached as high as –12.4%. (RA-5m). Again, the main reason for the LPM systems’ poor performance in these actions was likely due to the configuration of the Kalman filter, as noted previously [35, 36].

### Inter- and intra-unit reliability

The inter-unit reliability (as measured by CV) for a_max_ was in approximately the same range for both systems (GPS: CV 2.8 to 16.1%; LPM: CV 4.8 to 14.7%). The CV of a_max_ depended on the type of action and was highest for both systems during low accelerations from a standstill. Buchheit et al. [32] reported comparable results for the LPM system (CV ± 10.0% depending on the type of action) but worse results for the GPS (CV >10.0%). The GPS results of inter-unit reliability of this study are similar to those found by Lacome et al. [18], who calculated a CV of 6.4% for a_max_ during a 40 m sprint with a 16 Hz GPS.

The inter-unit reliabilities for both measurement systems were clearly better for v_max_ than for a_max_ and the CVs for v_max_ in every subcategory were within the recommended 5% from Hopkins [38]. The only exception was the RA-5m measured with the LPM system (CV of 5.4%). In contrast, Buchheit et al. [32] found that CV values of v_max_ measured by GPS tend to be higher than those of the LPM system, and are considerably higher than the ones measured in this study. As with a_max_, inter-unit CV measures of v_max_ for GPS are similar to those found by Lacome et al. [18] (CV of 0.5%), but better than those reported in studies by Coutts and Duffield [6] (CV 2.3 to 5.8% for different types of GPS devices) and by Johnston et al. [11] (CV of 8.1%; 15Hz GPS).

Interestingly, inter-unit reliability of a_max_ and v_max_ was generally better than intra-unit reliability for both measurement systems; the only exception to this was a_max_ in A-D with GPS. A possible explanation is that inter-unit reliability was determined simultaneously within the same trial (the RCC carried six devices for each system), whereas the intra-unit reliability was determined by reproducing the same type of action several times throughout the day. Furthermore, satellite availability for the GPS measurements most likely did not remain the same. However, the reason(s) for the low LPM intra-unit reliability are not clear. Different battery charging status is plausible but unlikely. Further research is thus needed to elucidate the factors that influence LPM systems’ intra-unit reliability.

### Comparison of local versus global positioning systems

Since LPM systems record with higher frequencies and their base stations are placed around a single site, it was assumed that measurements with the LPM systems would be more accurate than those with GPS. However, this hypothesis has not been confirmed. The validity of v_max_ and a_max_ depended strongly on the types of action and were similar for both the GPS and the LPM system in most of the team sport-specific actions that were analyzed. The only exceptions were found in the HA and RA-10m trials for v_max_, and RA-5m and RA-10m for a_max_ respectively, where GPS surprisingly outperformed the LPM system. The LPM system has significant potential to produce more accurate measurements of speed and acceleration. To benefit from the high potential of the LPM system, it is proposed that sport-specific Kalman filter configurations need to be integrated. Likewise, integrating inertial sensors, such as accelerometers, could allow for further improvements to both GPS and LPM system measurements.

### Study strengths and weaknesses

Due to the use of an RCC, the validity and inter- and intra-unit reliability of a_max_ and v_max_ could be determined without any interference from upper body movements, which have previously been shown to negatively affect the accuracy of laser measurements [16, 28–30]. Additionally, using a vehicle as a device carrier guaranteed laser measurements over a full soccer field without risking a loss of signal. Since the RCC runs on the steel ropes and a platform is attached on top of the RCC, six GPS and six LPM devices can record position measurements simultaneously and without obstructions in a horizontal position at shoulder height, simulating a player wearing the devices. This allowed us to compare the inter-unit reliability of multiple devices in the same trial. However, since the RCC mimicked team sport-specific actions on tensioned steel ropes, not all possible team sport-specific actions could be validated (e.g., acceleration after a COD with an angle of < 180°). However, all actions included in this study were very well recorded by a laser measurement setup.

The GPS and LPM systems’ biases were assessed in comparison to the laser system for each trial, and the results were then averaged to calculate the percentage bias. Thus, the laser system’s measurements were compared to GPS and the LPM system measurements for each trial, in order to avoid differences between trials caused by variation in the RCC’s operation, rather than the different measurement systems.

We are aware that the technology of position measurement systems is developing extremely fast and the recording frequency of GPS devices has been significantly increased in recent years (from 1 Hz to currently 18 Hz). Although position measurement systems with higher recording frequencies than the ones we have tested now exist, we suppose that the main results of this study can be nevertheless transferred to these systems. This is due to previous results showing that an increase in recording frequency did not automatically result in better data quality [11]. Measurement inaccuracies are likely due to the (wrong) filter configurations, rather than low recording frequencies. Furthermore, the LPM system does not make use of their integrated inertial sensors, although that might greatly improve the measurement accuracy, as they facilitate the differentiation and interpretation of various motor actions. We are convinced that these changes (appropriate filter settings, use of inertial sensors) would improve measurement accuracy to a greater extent than simply increasing the recording frequency, which currently seems to be the main target of the manufacturers.

## Conclusion

Although validity of v_max_ for both systems was generally good (except for LPM measurements during trials with CODs), validity of a_max_ was very low when measured by GPS, and, surprisingly, not higher when measured by the LPM system. The accuracy of a_max_ depended on the type of action, and was considerably lower when actions included any CODs. The Kalman filter configurations are the most likely explanation for this inaccuracy. Given our current knowledge of tracking system accuracy, it is debatable whether acceleration measurements should be included in game or training analyses.

However, acceleration measurements are fundamental to adequately describe physical loads in team sports, and improvements to Kalman filter properties are therefore necessary if team sports with different accelerations and CODs are to benefit from position-tracking systems. In our opinion, using the tested systems in team sports without filter improvements to measure accelerations during CODs is worthless due to the large measurement errors. Nevertheless, if teams do choose to include acceleration as an indicator of physical load, despite the recognized limitations in measuring it, it is essential that they interpret their results with caution.

## Supporting information

S1 Data (XLSX)

## References

[1] Akenhead R, Hayes PR, Thompson KG, and French D. Diminutions of acceleration and deceleration output during professional football match play. J Sci Med Sport. 2013; 16(6): 556–561. 10.1016/j.jsams.2012.12.005 23333009

[2] Bradley PS, Di Mascio M, Peart D, Olsen P, and Sheldon B. (2010). High-intensity activity profiles of elite soccer players at different performance levels. J Strength Cond Res. 2010; 24(9): 2343–2351. 10.1519/JSC.0b013e3181aeb1b3 19918194

[3] Gaudino P, Iaia FM, Alberti G, Strudwick AJ, Atkinson G, and Gregson W. Monitoring training in elite soccer players: systematic bias between running speed and metabolic power data. Int J Sports Med. 2013; 34(11): 963–968. 10.1055/s-0033-1337943 23549691

[4] Osgnach C, Poser S, Bernardini R, Rinaldo R, and di Prampero PE. Energy cost and metabolic power in elite soccer: a new match analysis approach. Med Sci Sports Exerc. 2010; 42(1): 170–178. 10.1249/MSS.0b013e3181ae5cfd 20010116

[5] Varley MC and Aughey RJ. Acceleration profiles in elite Australian soccer. Int J Sports Med. 2013; 34(01): 34–39. 22895869 10.1055/s-0032-1316315

[6] Coutts AJ and Duffield R. Validity and reliability of GPS devices for measuring movement demands of team sports. J Sci Med Sport. 2010; 13(1): 133–135. 10.1016/j.jsams.2008.09.015 19054711

[7] Frencken W, Lemmink K, and Delleman N. (2010). Soccer-specific accuracy and validity of the local position measurement (LPM) system. J Sci Med Sport. 2010; 13(6): 641–645. 10.1016/j.jsams.2010.04.003 20594910

[8] Ogris G, Leser R, Horsak B, Kornfeind P, Heller M, and Baca A. (2012). Accuracy of the LPM tracking system considering dynamic position changes. J Sports Sci. 2012; 30(14): 1503–1511. 10.1080/02640414.2012.712712 22906154

[9] Witte TH and Wilson AM. Accuracy of non-differential GPS for the determination of speed over ground. J Biomech. 2004; 37(12): 1891–1898. 10.1016/j.jbiomech.2004.02.031 15519597

[10] Hoppe MW, Baumgart C, Polglaze T, and Freiwald J. Validity and reliability of GPS and LPS for measuring distances covered and sprint mechanical properties in team sports. PLoS ONE. 2018; 13(2): e0192708. 10.1371/journal.pone.0192708 29420620 PMC5805339

[11] Johnston RJ, Watsford ML, Kelly SJ, Pine MJ, and Spurrs RW. Validity and interunit reliability of 10 Hz and 15 Hz GPS units for assessing athlete movement demands. J Strength Cond Res. 2014; 28(6): 1649–1655. 10.1519/JSC.0000000000000323 24276300

[12] Nikolaidis PT, Clemente FM, van der Linden CMI, Rosemann T, and Knechtle B. Validity and Reliability of 10-Hz Global Positioning System to Assess In-line Movement and Change of Direction. Front Physiol. 2018; 9: 228. 10.3389/fphys.2018.00228 29599725 PMC5862865

[13] Koklu Y, Arslan Y, Alemdaroglu U, and Duffield R. Accuracy and reliability of SPI ProX global positioning system devices for measuring movement demands of team sports. J Sports Med Phys Fitness. 2015; 55(5): 471–477. 25303067

[14] Waldron M and Highton J. Fatigue and Pacing in High-Intensity Intermittent Team Sport: An Update. Sports Med. 2014; 44(12): 1645–1658. 10.1007/s40279-014-0230-6 25047854

[15] Conte D. Validity of local positioning systems to measure external load in sport settings: a brief review. Hum Mov. 2020; 21(1).

[16] Akenhead R, French D, Thompson KG, and Hayes PR. The acceleration dependent validity and reliability of 10Hz GPS. J Sci Med Sport. 2014; 17(5): 562–566. 10.1016/j.jsams.2013.08.005 24041579

[17] Buchheit M, Al Haddad H, Simpson BM, Palazzi D, Bourdon PC, Di Salvo V, et al. Monitoring accelerations with GPS in football: time to slow down? Int J Sports Physiol Perform. 2014; 9(3): 442–445. 10.1123/ijspp.2013-0187 23916989

[18] Lacome M, Peeters A, Mathieu B, Bruno M, Christopher C, and Piscione J. Can we use GPS for assessing sprinting performance in rugby sevens? A concurrent validity and between-device reliability study. Biol Sport. 2019; 36(1): 25–29. 10.5114/biolsport.2018.78903 30899136 PMC6413573

[19] Aughey RJ. Applications of GPS Technologies to Field Sports. Int J Sports Physiol Perform. 2011; 6(3): 295–310. 10.1123/ijspp.6.3.295 21911856

[20] Barbero-Alvarez JC, Coutts A, Granda J, Barbero-Alvarez V, and Castagna C. The validity and reliability of a global positioning satellite system device to assess speed and repeated sprint ability (RSA) in athletes. J Sci Med Sport. 2010; 13(2): 232–235. 10.1016/j.jsams.2009.02.005 19446495

[21] Sonderegger K, Tschopp M, and Taube W. The Challenge of Evaluating the Intensity of Short Actions in Soccer: A New Methodological Approach Using Percentage Acceleration. PLoS ONE. 2016; 11(11): e0166534. 10.1371/journal.pone.0166534 27846308 PMC5112910

[22] Haugen T and Buchheit M. Sprint Running Performance Monitoring: Methodological and Practical Considerations. Sports Med. 2016; 46(5): 641–656. 10.1007/s40279-015-0446-0 26660758

[23] Malone JJ, Lovell R, Varley MC, and Coutts AJ. Unpacking the Black Box: Applications and Considerations for Using GPS Devices in Sport. Int J Sports Physiol Perform. 2017; 12(s2): s2-18–s2-26. 10.1123/ijspp.2016-0236 27736244

[24] Varley MC, Fairweather IH, and Aughey RJ. Validity and reliability of GPS for measuring instantaneous velocity during acceleration, deceleration, and constant motion. J Sports Sci. 2012; 30(2): 121–127. 10.1080/02640414.2011.627941 22122431

[25] Harrison AJ, Jensen RL, and Donoghue O. A Comparison of Laser and Video Techniques for Determining Displacement and Velocity During Running. Meas Phys Educ Exerc Sci. 2005; 9(4): 219–231.

[26] Tuerk-Noack U and Schmalz T. Field trial of the LAVEG laser diode system for kinematic analysis in various kinds of sports. Int Symp Biomech Sports (ISBS); 1994.

[27] Duthie GM, Pyne DB, Marsh DJ, and Hooper SL. Sprint patterns in rugby union players during competition. J Strength Cond Res. 2006; 20(1): 208–214. 10.1519/R-16784.1 16506864

[28] Bezodis NE, Salo AI, and Trewartha G. Measurement error in estimates of sprint velocity from a laser displacement measurement device. Int J Sports Med. 2012; 33(06): 439–444. 10.1055/s-0031-1301313 22450882

[29] Orendurff MS, Segal AD, Klute GK, Berge JS, Rohr ES, and Kadel NJ. The effect of walking speed on center of mass displacement. J Rehabil Res Dev. 2004; 41(6): 829–834. 10.1682/jrrd.2003.10.0150 15685471

[30] Schache AG, Blanch P, Rath D, Wrigley T, and Bennell K. Three-dimensional angular kinematics of the lumbar spine and pelvis during running. Hum Mov Sci. 2002; 21(2): 273–293. 10.1016/s0167-9457(02)00080-5 12167303

[31] Duffield R, Reid M, Baker J, and Spratford W. Accuracy and reliability of GPS devices for measurement of movement patterns in confined spaces for court-based sports. J Sci Med Sport. 2010; 13(5): 523–525. 10.1016/j.jsams.2009.07.003 19853507

[32] Buchheit M, Allen A, Poon TK, Modonutti M, Gregson W and Di Salvo V. Integrating different tracking systems in football: multiple camera semi-automatic system, local position measurement and GPS technologies. J Sports Sci. 2014; 32(20):1844–1857. 10.1080/02640414.2014.942687 25093242

[33] Waldron M, Worsfold P, Twist C, and Lamb K. Concurrent validity and test-retest reliability of a global positioning system (GPS) and timing gates to assess sprint performance variables. J Sports Sci. 2011; 29(15): 1613–1619. 10.1080/02640414.2011.608703 22004326

[34] Nagahara R, Botter A, Rejc E, Koido M, Shimizu T, Samozino P, et al. Concurrent Validity of GPS for Deriving Mechanical Properties of Sprint Acceleration. Int J Sports Physiol Perform. 2017; 12(1): 129–132. 10.1123/ijspp.2015-0566 27002693

[35] Kalman RE. A New Approach to Linear Filtering and Prediction Problems. J Basic Eng. 1960; 82: 35–45.

[36] Stevens TGA, de Ruiter CJ, van Niel C, van de Rhee R, Beek PJ, and Savelsbergh GJP. Measuring Acceleration and Deceleration in Soccer-Specific Movements Using a Local Position Measurement (LPM) System. Int J Sports Physiol Perform. 2014; 9(3): 446–456. 10.1123/ijspp.2013-0340 24509777

[37] Fischer-Sonderegger K, Taube W, Rumo M, and Tschopp M. Measuring Physical Load in Soccer: Strengths and Limitations of 3 Different Methods. Int J Sports Physiol Perform. 2019; 14(5): 627–634. 10.1123/ijspp.2017-0768 30427245

[38] Hopkins W. G. Measures of reliability in sports medicine and science. Sports Med. 2000; 30(1): 1–15. 10.2165/00007256-200030010-00001 10907753

