# Supplementary material for: How far from the gold standard? Comparing the accuracy of a Local Position Measurement (LPM) system and a 15 Hz GPS to a laser for measuring acceleration and running speed during team sports
Source: PLoS One. 2021 Apr 23;16(4):e0250549. doi: 10.1371/journal.pone.0250549 (PMC8064559; doi:10.1371/journal.pone.0250549)
Supplement: Attachment — Submitted filename: Response to Reviewers.pdf [file pone.0250549.s002.pdf]

Dear Editor
Dear reviewers

Thank you for giving us the opportunity to revise our manuscript. We are thankful to the reviewers for the time and valuable comments on our manuscript, which have helped us "tighten" and improve the quality of our paper.

<u>Response to reviewers</u>

Reviewer #1:
Introduction
The introduction is quite long and needs some adjustment. Overall, authors should consider that introduction should lead the reader to understand the current literature on the selected topic and the rationale for the study. In this section as it stands there are many indications about the methods used in the study as well as what it has been done in this study. Please re-consider this part taking also into considerations the following suggestions:

> Thank you for this feedback. We have deleted some parts of the introduction which focus on the method used, in order to concentrate on the essentials of the instructions and to shorten it.

Line 49: it is necessary referencing the first sentence.

> Done.

Line 51-62: Also here are present too many general information that should be sustained by the use of references.

> Done.

Line 74: I would suggest avoiding the use of questions which is more colloquial and not fully suitable in a scientific paper.

> Thank you for your remark. I deleted this question in the paper.

Line 89-95: this part should be deleted from introduction since it applies to method section.

> Done.

Line 117: "Our investigation is the first to…" This applies to a possible discussion section rather than introduction.

> Done.

Methods
Line 138: why different number of trials were assessed for each sub-category? Please specify.

> If several accelerations occur in one sport-specific movement pattern, all accelerations were assigned to their respective sub-categories. This increases the number of trials within the affected sub-categories only. Thus, not all sub-categories have the same number of trials. E.g. a movement pattern with two accelerations interspersed with an abrupt deceleration can account for both, low acceleration from standstill [LA] and acceleration after an abrupt deceleration [A-D].
>
> We have adjusted the text in the article as followed:
> "One team sport-specific movement pattern can provide several team sport-specific actions (e.g. a movement pattern with two accelerations interspersed with an abrupt deceleration can be

accounted for both low acceleration from standstill [LA] and acceleration after an abrupt deceleration [A-D]). Team sport-specific actions differed in acceleration capacity, initial speed, maximal speed, number of CODs, and were divided into seven subcategories. In order to present a number of trials within each subcategory (Table 1), sport-specific movement patterns were carried out several times."

Table 1: how the categories were identified? For instance, based on what an acceleration was considered low medium or high? Much more information is required about this categories' selection and in general about the methods applied. For instance, on which distances were measured, which accelerations and decelerations were adopted etc.

We made changes/adjustments to the paper, based on your comments.

Adjusted details in the paper:
"Accelerations occurred between 0 and 7.8 $m \cdot s^{-2}$, and were classed as low acceleration if < 3.5 $m \cdot s^{-2}$ or high if > 3.5 $m \cdot s^{-2}$. Flying start accelerations were initialized from speeds between 6 and 15 $km \cdot h^{-1}$."
…
"The steel ropes were stretched across the entire length of the soccer field through the middle of the field. The actions were carried out in the middle of the field."

Results

Line 210: abbreviations should be expressed in the text and not with reference to table. Please change accordingly.

Done.

Discussion

This section is well-written and easy to follow. I would just suggest to include the limitations of the study and some practical applications for the use of GPS and LPS.

Based on your feedback, we have adjusted the chapter "Study strengths and weaknesses". Practical applications are listed under the adapted "Conclusions".

Reviewer #2:

General Comments:

The current paper investigated the validity, inter and intra-unit reliability of Local and Global position measurement systems compared to laser measurements. The paper is generally, well written with few editorial concerns (see Specific Comments). However, there is a major problem in why the authors used a filtering technique that they acknowledge was most likely inappropriate for the data (see Specific Comments). Furthermore, in this reviewer's opinion, they should have been more forceful in their Conclusion by stating the unsuitability of using these devices for determining acceleration during change of direction. As this type of movement is extremely common in sport (as noted by the authors) and the error so large, the methodology is of virtually no use.

Thank you for your feedback. We would like to express our thoughts on the filter settings used in the paper.

Indeed, the filter settings are a major problem in position measurement systems. This problem has already been indicated by other studies, however, no study shows the extent of the problem/error in different team sport-specific actions.

The aim of the study was to measure and compare two <u>commercially available</u> products in the field. We examined the validity, inter- and intra-unit reliability of these devices, as they were

sold to and are used by clubs. It was not the aim of the study or the intention of the authors to improve the validity of the devices.

This paper shows the manufacturers of these products the large measurement errors in acceleration (especially when changing direction), with the prospect, that they come to the correct conclusions (i.e. making improvements in the filter settings, as written in the conclusion). Furthermore, this paper should sensitize scientists in the field and (fitness) coaches to the large measurement errors when using one of these systems. We agree with reviewer #2 that using these devices for determining acceleration during change of direction is unsuitable. Therefore, the last sentence in the conclusion has been adjusted accordingly.

Specific Comments:

Line 31-35: Somewhat a run on sentence, which I would suggest breaking apart as follows: "Mean percentage biases (MPBs) of maximal acceleration (amax) and maximal running speed (vmax) were used to measure validity, and mean between-device. Mean within-device standard deviations of the percentage biases (bd-SD and wd-SD) of amax and vmax were used to measure inter- and intra-unit reliability, respectively."

Thank you for that valuable comment. I have adjusted the sentences as suggested.

Lines 92-95: What about making the car turn? Although it would not be an exact diagonal movement, it could simulate that sort of change. Even if this is an inappropriate type of movement (i.e. rounding off the direction), the authors should probably address it.

Thank you for your feedback. We would like to highlight our thoughts on the method used in the paper. Carrying out the test with a remote-controlled car that drives over a football field on two steel ropes is a very complex and difficult test setting, which has many advantages: 1) its acceleration phases can be precisely determined with a laser (in contrast to human acceleration phases); 2) laser measurements can be guaranteed over a full soccer field without risking a loss of signal; 3) several devices could be worn on the same height as when athletes do the test; 4) several devices can easily be attached to the car to record data simultaneously, making it especially valuable as a measure of inter-unit reliability; and 5) all antennas of the LPM System are in a horizontal position as requested.

However, choosing this already complex test setting with an RCC on two steel ropes, tightly stretched over a soccer field with two tractors, does not solve all problems. Thus, turning the car on the steel ropes or doing COD with angles smaller than 180 were not possible.

Line 111: Suggest inserting "as a device carrier."

Done.

Line 208: The term "MPB" is not defined until Line 225-226. While "MPBs" is defined in the abstract, it should be here as well. Please correct.

Done.

Line 210: Please capitalize "Table".

Done.

Table 2: Nearly all of the CVs are above 5%, a level that Hopkins suggests renders the performance near useless when considering repeatability (http://www.sportsci.org/resource/stats/index.html). Therefore how practical is it to use these devices for determining acceleration? As that is one of the objectives of the current study, how can the authors suggest the Methods are valid/reliable? (Please see comment in the Conclusion)

Indeed, as the reviewer #2 correctly noted, the acceleration measurements with these two tested position measurement systems are often above the 5% level (CV> 5%). Therefore, knowing these results, measuring accelerations in team sports is actually questionable. However, it is a fact that GPS and / or LPM systems are often used by many (soccer) clubs, regardless of the (in)accuracy of the measurements, often without considering the accuracy of the measurement systems. The aim of this work was therefore, to show the accuracy of the systems and, as we have now reinforced in the conclusion, to emphasize that recording physical load by acceleration measurements (especially for units with many changes of direction) should not be done with these two systems (without improving the filter settings).

See also the answer to the “general comment” at the beginning.

Line 222: Is this sentence supposed to be a footnote to the Table? If so, I'd suggest indenting it. Suggest doing the same for Line 254.

Done.

Lines 232-234: This reviewer would suggest a bit more explanation as to the "combination" referred to here. I understand that it appears to just be summing them, but why is this useful. An explanation would make it more clear to the reader.

Thank you for the remark. We have adjusted the text in the article as followed:

“If a GPS or LPM device is randomly chosen for repeated measures, the whisker illustrates the percentage measurement error.”

Lines 246-247: Do the authors have any explanation for this variation? There doesn't appear to be anything noted in the Discussion.

An explanation would be purely speculative and cannot be made on the basis of the results. This study only describes the facts. As described in the next paragraph, if manufacturers take the right steps to improve the system based on our results, a major aim will have been achieved.

Lines 312-316: As the authors were aware of this limitation, why not try a different type of filter? Would it not be possible to use the raw data and do the filtering outside of the device if the filter system was within the device? If this was impossible, then the authors should state why this approach was not used.

Thank you for your comment. This study shows the accuracy of two different systems (GPS and LPM) which are in use by many team sport clubs in the exact same way as they were tested in this study. The aim of the study was to validate two commercially available positioning measurement systems without changing filter properties (and not to improve the commercially available products). If, with this study, manufacturers can be asked to improve their filter settings and, at the same time, to sensitize users to the fact that the accuracy depends on the action measured, the goal has been achieved. Changes to the kalman filter are only possible by the manufacturer and not by the end-users. Nevertheless, it would be interesting to use the raw data and do the filtering outside of the device in the future.

Lines 339-340: Please provide some explanation as to why you would make this assumption.

As described in the lines 328-332 (Manuscript), the speed never reached 0 during the CODs, even though the effective speed was 0 for a few tenths of a second. When the COD angle is smaller than 180 degree (e.g. 100), the speed is never 0 and therefore we assume, that the prediction of the next position and the prediction of the speed direction is more accurate and therefore the measurement error for acceleration smaller. With our test setting, a lot of limitations could be eliminated which existed in previous studies with human beings or with a

vehicle on the floor (see comments above). However, some limitations still exist (no COD with smaller angles than 180 could be assessed). Because we did not test COD with smaller angles than 180, we deleted the assumption in the text.

Lines 365-367: While the GPS CVs were almost 10 times lower than LPM, is this really a major issue? Both are below the values recommended by Hopkins for CV (except for RA-5, which is just over 5%), so maybe it doesn't really matter...

You are right. Thank you for that comment. We have adjusted the text in the article accordingly.

Lines 435-436: I would go even further than this caveat. The method should NOT be used for COD, where the error was so high as well as lagging behind.

You are right. Thank you for that remark.

We hope that this article will alert both the manufacturers of the products and the people responsible for the system in the clubs so that the received data can be viewed with the necessary scepticism. As the results show, the acceleration measurement with COD is indeed very imprecise, so your objection is certainly justified. The now additionally supplemented sentence in the conclusion clearly highlights this problem.

We thank you for your time and look forward to your response.

Yours sincerely,

Karin Fischer-Sonderegger, Wolfgang Taube, Martin Rumo, Markus Tschopp

Corresponding author: Karin Fischer-Sonderegger, Swiss Federal Institute of Sport Magglingen SFISM, Section for Elite Sport, 2532 Magglingen, Switzerland, email: karin.fischer@baspo.admin.ch
