# Supplementary material for: How far from the gold standard? Comparing the accuracy of a Local Position Measurement (LPM) system and a 15 Hz GPS to a laser for measuring acceleration and running speed during team sports
Source: PLoS One. 2021 Apr 23;16(4):e0250549. doi: 10.1371/journal.pone.0250549 (PMC8064559; doi:10.1371/journal.pone.0250549)
Supplement: Attachment — Submitted filename: Response to Reviewers.pdf [file pone.0250549.s003.pdf]

Dear Editor
Dear Reviewers

With this letter, we will submit the revised manuscript (PONE-D-20-09964R1) entitled, "How far from the gold standard? Comparing the accuracy of a local position measurement (LPM) system and a 15 Hz GPS to a laser for measuring acceleration and running speed during team sports".
We would like to thank the reviewers for the careful and constructive reviews. It is our belief that the manuscript is substantially improved after making all the suggested edits. Following this letter are the reviewer comments with our responses in italics, including how the text was modified. The revision has been developed in consultation with all coauthors, and each author has given approval to the final form of this revision.

<u>Response to reviewers</u>
Reviewer #1:
The authors addressed all my previous comments improving the quality of the manuscript. I would just suggest the addition of this reference, which would be fully suitable in their manuscript:

Thank you for your remark. We integrated the proposed paper.

Reviewer #2:
General Comments:
The current version of the manuscript is much improved. There remain a few editorial issues (see Specific Comments), but otherwise only one area of content that should be addressed. I would encourage the authors to provide some explanation for why the validity of acceleration was so different when there were multiple accelerations (see Specific Comments). While they have previously stated their reluctance to speculate on outcomes (an admirable trait), they could still provide a possible explanation, which would provide readers with a basis for what should be examined further.

Done. Note the comment at line 344-346.

Specific Comments:
Lines 112-113: Single paragraph sentences should be avoided, if possible. That could be accomplished easily by just combining these two paragraphs. The second is really just carrying on from the first anyway.

Done

Line 127: Suggest replacing "differed" with "differing".

Done

Line 154: Maybe insert "by the device's software". This would assist in indicating that you used the software of the device manufacturer, and didn't filter the data any further.

Done

Lines 169-170: I still have a problem with "linear and multi-directional" being used to describe the movements. Although the direction changed 180 degrees, it was still linear to my way of thinking; as opposed to the "turning" aspect mentioned in the first review. Can the authors come up with a different word for "multi-directional"? I'd encourage that if it were possible. Maybe "forward and backward" or something like that...

Done. I have adjusted the sentences as suggested:
"Thus, the aim of this study was to determine and compare the validity of commercially available GPS and LPM systems in assessing acceleration and speed during forward and

backward and single directional actions, using a laser measurement system as gold standard."

Lines 223-229: It is not clear why there are bolded phrases in these lines. If they are to indicate where the figures go, those lines should be separated out from the text, as was done for Figure 1. Otherwise, this section is awkward and difficult to follow what the authors are getting at here.

Yes, these lines should be separated out from the text. We have adjusted the text in the article as followed:

"Fig 2A and Fig 2B. Validity, inter- and intra-unit reliability of maximal acceleration (a_{max}; Fig 2A) and maximal speed (v_{max}; Fig 2B).

Mean percentage bias (MPB; relative bias of the GPS and LPM devices compared to the laser) of a_{max} and v_{max} of 7 different team sport-specific actions indicates the validity of a_{max} and v_{max}. The first whisker of each subcategory within the black and grey squares is the between-device SD of the percentage biases of a_{max} and v_{max}, and represents the inter-unit reliability of a_{max} and v_{max}. The second whisker is the within-device SD of the percentage biases of a_{max} and v_{max}, and represents the intra-unit reliability of a_{max} and v_{max}. The third whisker of each subcategory is the combination of the between- and within-device SD of the percentage biases of a_{max} and v_{max}, which indicates the inter- and intra-unit reliability of a_{max} and v_{max}. If a GPS or LPM device is randomly chosen for repeated measures, the third whisker illustrates the percentage measurement error."

Lines 233-236: These lines appear to be combined sentences that don't match up. The version in the tracked changes seems to read correct, but this one does not. I think the words as follows need to be removed: "The summarized reliability illustrates the percentage measurement error if a GPS or LPM device is randomly chosen for repeated measures."

Thank you for your remark. We have corrected the mistake.

Line 318: Typo "smoothed"

Done

Lines 342 & 344: Suggest inserting a comma after "RA-5m". It helps to clarify that you are referring to how A-D differs from all three of the other conditions "A-COD, RA-5m, and RA-10m".

Done

Lines 344-346: Could the authors suggest a possible reason as to why this difference may have occurred? Perhaps it was due to the multiple accelerations that took place in the A-COD, RA-5m, and RA-10m. This is somewhat alluded to in lines 340-342, where the authors state "In addition, when acceleration occurred after an immediate deceleration but without a COD (A-D), the validity of amax was noticeably better than in trials with one or more CODs (A-COD, RA-5m and RA-10m)". Is it possible that the multiple accelerations were "averaged" or the effect was additive? While the authors may not have data to explicitly state this, they could note that additional work could be done to examine this possibility.

Thank you for that comment. We have implemented a possible explanation in the paper:

"One possible explanation for the large difference in validity of a_{max} between A-D and A-COD, RA-5m, and RA-10m could be due to the Kalman filter settings. In contrast to A-COD, RA-5m, and RA-10m, the direction of A-D is already correctly predicted by the Kalman filter. This leads to the difference in validity between a stop-and-go with COD (A-COD, RA-5m and RA-10m) compared to without COD (A-D). However, this can only be speculated and would have to be verified in further studies with different Kalman filter settings.".

Line 406: Suggest replacing "on" with "at".

Done
